# Negative-pressure wound therapy in thoracic and abdominal surgery: meta-analysis of randomized trials

**DOI:** 10.1093/bjsopen/zrag027

**Published:** 2026-05-20

**Authors:** Adil S Lakha, Salma Neves, Younis Alemour, Hannah McGivern, Alex Gordon-Weeks

**Affiliations:** Nuffield Department of Surgical Sciences, University of Oxford, Oxford, UK; Department of Hepatobiliary and Pancreatic Surgery, Oxford University Hospitals NHS Foundation Trust, Oxford, UK; Thames Valley Foundation School, Oxford, UK; Bodleian Healthcare Libraries, University of Oxford, Oxford, UK; Department of Integrative Biology, Sorbonne University, Paris, France; Nuffield Department of Surgical Sciences, University of Oxford, Oxford, UK; Department of Hepatobiliary and Pancreatic Surgery, Oxford University Hospitals NHS Foundation Trust, Oxford, UK

## Abstract

**Background:**

Around 30 000 patients undergo emergency laparotomy in the UK each year, and a similar number of patients undergo open cardiothoracic surgery. Surgical site infection is a common complication associated with increased morbidity, prolonged hospital stay, and higher healthcare costs. Negative-pressure wound therapy has been proposed as a prophylactic strategy to reduce wound complications, but trial evidence has been inconsistent.

**Methods:**

This systematic review and meta-analysis was carried out using PRISMA guidelines and was registered prospectively in PROSPERO (CRD420251010516). A literature search was carried out in March 2025 (updated December 2025), and titles and abstracts were screened against predefined inclusion criteria. Trials assessing patients undergoing open thoracic or abdominal surgery for any indication in adult patients assessing the risk of surgical site infection as an outcome were included. Quality assessment was performed using Cochrane’s risk-of-bias 2 tool. Summary statistics for outcomes of interest underwent meta-analyses to a confidence interval of 95% and are presented as forest plots.

**Results:**

Some 12 427 patients across 45 randomized trials in abdominal and thoracic surgery were included for analysis. Negative-pressure wound therapy significantly reduced surgical site infection compared with standard dressings (odds ratio (OR) 0.53, 95% confidence interval 0.42 to 0.66). The effect was consistent across commercial devices (PICO™ and Prevena™). Negative-pressure wound therapy was associated with shorter hospital stay (mean difference −1.67 (95% confidence interval −3.19 to −0.16) days), but not with reduced risk of organ/space infection (OR 0.92, 0.67 to 1.25), wound dehiscence, or reoperation. Only three studies included thoracic surgery and no significant difference in surgical site infection was found (OR 0.44, 0.00 to 45.25). Publication bias was detected; trim-and-fill analysis attenuated but did not eliminate the benefit (adjusted OR 0.70, 0.54 to 0.90). Adverse events and patient-reported outcomes were reported infrequently, and showed no consistent differences.

**Conclusion:**

Negative-pressure wound therapy was associated with a nearly 50% reduction in SSI and shorter hospital stay after open abdominal surgery, with consistent benefit across device types. However, evidence of publication bias, and limited long-term and patient-reported outcome data suggest that effect size may be overestimated. Selective use in high-risk patients is supported.

## Teaser

This systematic review and meta-analysis of 45 randomized trials including over 12 000 patients found that closed-incision negative-pressure wound therapy (NPWT) significantly reduces surgical site infections (SSIs) and shortens hospital stay following open abdominal surgery, with consistent effects across device types. No benefit was observed for organ/space infection, fascial dehiscence or reoperation, and evidence of publication bias suggests that, although the magnitude of benefit may be overestimated, the reduction in SSI remains statistically significant when adjusted for this. These findings support selective use of NPWT in high-risk patients, while underscoring the need for cost-effectiveness analyses and long-term outcome data.

## Introduction

Almost two million patients undergo laparotomy in the USA each year, and hundreds of thousands undergo sternotomy for various indications, in both the emergency and elective setting^1,2^. These incisions provide surgical access for major thoracic and abdominal surgery. Following a procedure, the wounds require closure, and various techniques have been described to close the thoracic and abdominal wall in layers, to promote wound healing and minimize complications^3,4^. One such adjunct to closure of these wounds is the use of negative-pressure wound therapy (NPWT), which aims to obliterate the dead space in the subcutaneous layers, thus reducing the volume of serous fluid build-up under the skin surface. In theory, this reduces the chance of infection developing at the surgical site.

Surgical site infection (SSI) can be associated with significant morbidity, and may result in wound dehiscence necessitating further surgery, systemic illness such as sepsis, or future risk of incisional hernia^5^. SSIs are one of the top five healthcare-acquired infections, accounting for approximately one-third of the $9.8 billion cost to the US healthcare system, increasing average length of hospital stay by 9.58 days at an additional cost of $38 656^6,7^. Several risk factors exist for the development of SSIs. Patient-related factors include smoking, diabetes, immunosuppression, obesity, and malnutrition^8^. Recognized operative influences include emergency surgery, degree of contamination, revisional surgery, and soft tissue trauma^8,9^.

NPWT has been evaluated in a growing number of randomized clinical trials (RCTs) across surgical specialties. Although early studies suggested potential reductions in SSI and wound complications, results have been inconsistent, and previous meta-analyses have been limited by small sample sizes, heterogeneous inclusion of observational designs, or restriction to single surgical specialties^10–14^. Furthermore, questions remain regarding the magnitude of benefit, device-specific efficacy, applicability across surgical populations, and cost-effectiveness in routine practice.

Given the increasing volume, complexity, and multimorbidity of patients presenting for major open thoracic and abdominal surgery, there is a growing need to prevent the development of wound complications, surgical morbidity, and healthcare resource utilization^15^. This systematic review and meta-analysis therefore aimed to assess the efficacy of NPWT in patients undergoing open thoracic or abdominal surgery with closed wounds, providing a comprehensive overview of its application and utility in a pan-specialty manner.

## Methods

This systematic review was conducted using guidance from the *Cochrane Handbook for Systematic Reviews of Interventions*^16^. These findings are reported according to the search extension for PRISMA guidelines^17^. A study protocol was developed on 13 March 2025 before starting this systematic review of the literature concerning the use of NPWT on SSI, with particular focus on RCTs. The protocol was registered with PROSPERO (CRD420251010516) in June 2025^18^.

### Search strategy

MEDLINE and Embase on Ovid, as well as the Cochrane Central Database of Controlled Trials, Web of Science Core Collection and Scopus, were searched on 18 March 2025 (last updated 1 December 2025). Each of these databases was searched separately using a combination of free-text keywords and subject headings or Medical Subject Headings terms which were similar to, and adapted from, a search in Ovid MEDLINE (*Table S1*). The search strategies for each database can be found in *supplementary methods* and *Tables S1–S3*.

**Fig. 1:**
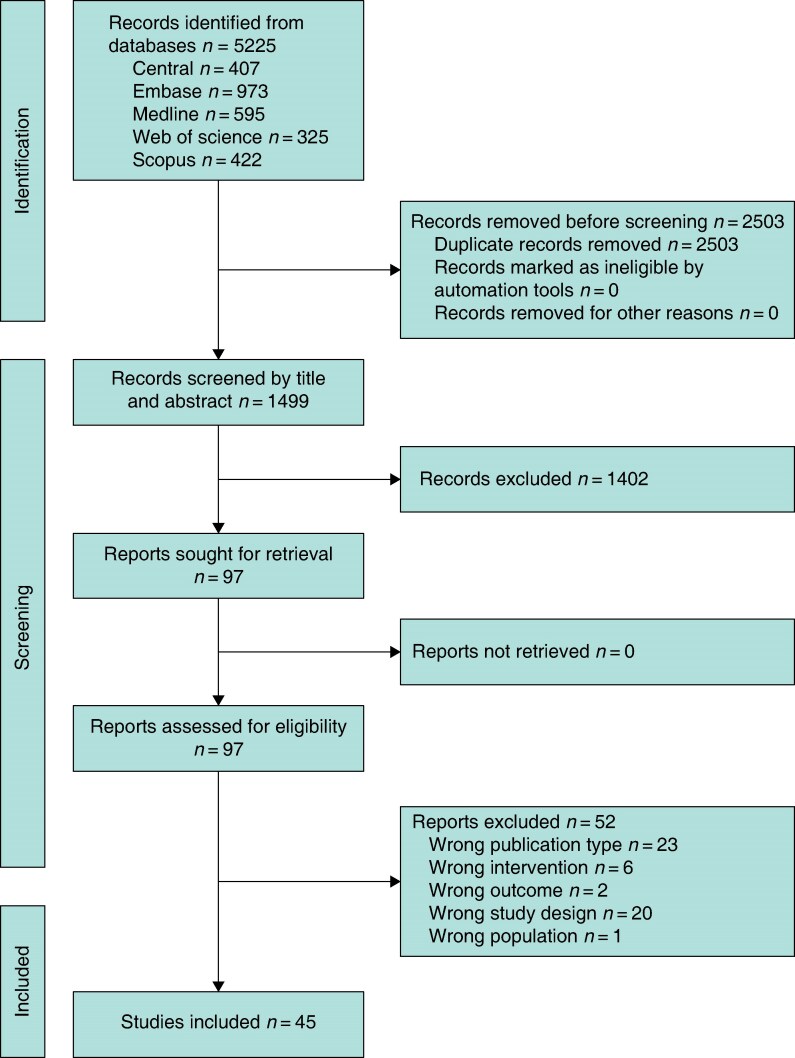
PRISMA flow chart showing selection of articles for review

The total number of records for each database search is summarized in *Fig. 1*. Search filters for RCTs were applied to each search, excluding the search in the Cochrane Central Database of Controlled Trials as it is not recommended to do so^16^. The RCT filters for MEDLINE and Embase were adapted from search filters reported in the *Cochrane Handbook for Systematic Reviews of Interventions*^16^. Published search filters were also applied to the search strategies for Web of Science and Scopus. Any additional limits were applied thereafter using the inclusion and exclusion criteria set out in the protocol. All search strategies were devised and run by the information specialist (H.M.) for this systematic review only and have not been used elsewhere previously. The Ovid MEDLINE search strategy was reviewed and approved by the main author (A.L.) before searches being run on each database.

To deduplicate, all records were exported from each database as Research Information Systems files and uploaded to EndNote™ 21.2 (Philadelphia, PA, USA). The deduplication function in EndNote was used to identify duplicate records. The deduplication process was run several times, with different edited preferences according to steps outlined by Bramer *et al*.^19^. After each subsequent step, each set of duplicate results was examined carefully to identify any false duplicates and ensure that these were not removed.

### Article screening

Each article was assessed by two separate authors using the inclusion criteria outlined below, and any disagreement regarding the eligibility of an article was discussed. Agreement was reached by consensus with a third, independent, reviewer. There were no language or time-period restrictions. Abstract-only publications, editorials, letters, commentaries, case reports/case series with fewer than ten patients, and conference presentations were excluded, as were retrospective studies and non-randomized cohort studies. RCTs that compared wound-related outcomes of patients who underwent open thoracic or abdominal surgery for any elective or emergency indication where the wound was closed primarily were included. Control groups included patients without NPWT, in whom simple wound dressings only were used, such as dry sterile gauze, hydrocolloid, honeycomb or any other standard surgical wound cover. The authors of studies that met the inclusion criteria, but reported ambiguous or missing data for either the population or outcomes of interest, were contacted for further clarification before exclusion.

### Quality assessment

The Cochrane risk-of-bias 2 tool^20^ for RCTs was used to evaluate relevant studies identified. Two independent reviewers undertook quality assessment. Any discrepancies were discussed with a third author until consensus was reached. Results are presented in both the standardized format and as a weighted bar plot to aid visualization.

### Data extraction

Data were extracted by two independent authors for each paper using a standardized and predesigned data collection form. Data were extracted, where available, on study design characteristics, cohort information including proportion of patients undergoing elective *versus* emergency surgery, information on underlying surgical pathology, relevant co-morbidities such as smoking, body mass index (BMI), immunosuppression, concurrent antibiotic therapy, hypoalbuminaemia, wound contamination, and presence of diabetes. Outcomes of interest including SSIs, wound dehiscence, Clavien–Dindo complication grade^21^, postoperative pain, quality of life (QoL) scored using any validated tool, incisional hernia risk, and length of hospital stay, were also recorded, when reported. Data were recorded on an intention-to-treat basis to preserve the effects of randomization.

### Data synthesis

Data analyses were undertaken and figures extracted from Microsoft^®^ Excel (Microsoft, Redmond, WA, USA) and the statistical package RevMan version 5.8.0 (Cochrane Collaboration (London, UK)), and further analyses undertaken in R version 4.5.2 for MacOS™ (R Project for Statistical Computing, Vienna, Austria). Funnel plots were used to assess publication bias in studies that reported the primary outcome of interest, and analysed for asymmetry by means of Egger’s linear regression, using a mixed-effects meta-regression model, and standard error as the predictor^22^. The trim-and-fill method was used to adjust for missing studies using the inverse-variance method with the L-estimator, and restricted maximum-likelihood estimator for τ. Heterogeneity was assessed for the meta-analyses using the *I*^2^ statistic (< 20%, low; 20–40%, moderate; > 40%, high), with the Mantel–Haenszel method and a random-effects model used because of the high expected heterogeneity between the studies included in the meta-analysis. Prediction interval was based on t-distribution (53 degrees of freedom). Summary statistics used to characterize the distribution of prespecified dichotomous outcomes of interest underwent meta-analyses to a confidence interval of 95% and are presented as forest plots for odds ratios (ORs)^23^. For continuous outcomes, the pooled effect size was expressed as the mean difference with a 95% confidence interval, calculated using the inverse-variance method.

For the purposes of meta-analysis, the work of Costa *et al*.^24^ was considered as two separate trials, as this study included two intervention arms (control, Prevena™, PICO™), and so outcomes for both types of vacuum-assisted therapy (compared with control) were recorded separately. Fisher’s exact test and Pearson’s χ^2^ test with Yates’ continuity correction were used to compare categorical variables in order to assess clinicopathological comparability of randomized cohorts within each included study^25^. Baseline characteristics are, however, reported without formal statistical comparison, in line with item 15 of CONSORT and to avoid α inflation in multiple testing, leading to potentially falsely significant results^26,27^.

## Results

**Fig. 2:**
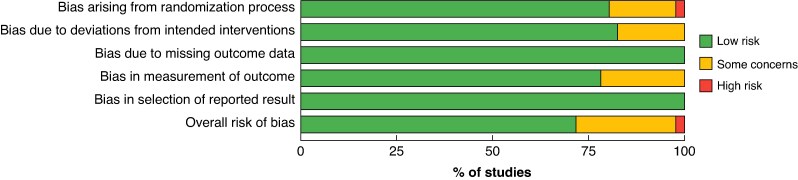
Risk-of-bias weighted bar plot

A total of 45 RCTs^10,28–68^ involving 12 427 patients was included (*Fig. 1* and *Table S4*). The trials spanned from 2010 to 2025, and were conducted across Europe, North America, Asia, and Australasia. The majority of trials enrolled patients undergoing major abdominal surgery, including colorectal, hepatopancreatobiliary, bariatric, and obstetric procedures; a smaller number investigated cardiac surgery. Most trials compared NPWT with standard gauze or adhesive dressings; PICO™ and Prevena™ were the most frequently studied commercial systems (63% of studies). Follow-up was typically 30 days, and longer follow-up beyond 90 days was rare. Thirty-three trials were rated as being at low risk of bias, with only one^24^ rated as high risk owing to concerns with the randomization process (*Fig. 2* and *Fig. S1*).

Baseline characteristics of patients included in control and intervention arms were similar, including important risk factors for wound complication such as smoking, diabetes, and obesity (*Table S5*). Where reported, immunosuppression was similar across groups, as was hypoalbuminaemia, except in one study (AbdelDayam *et al*.) that reported significantly higher rates of low albumin level in the intervention group compared with the control group (*P* = 0.001).

### SSI

**Fig. 3:**
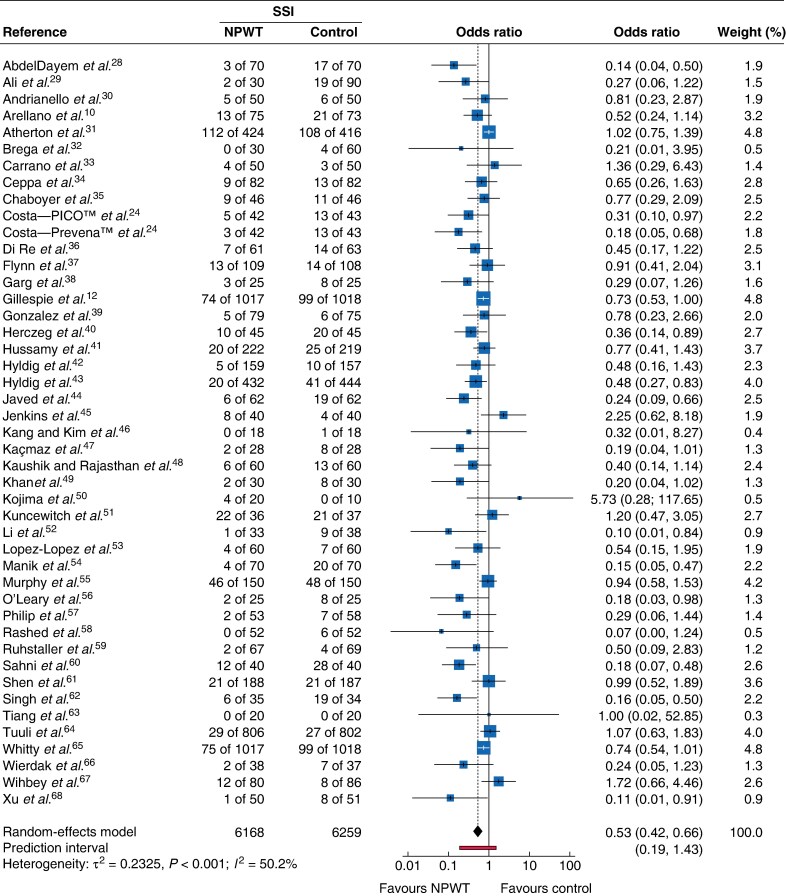
Forest plot showing meta-analysis of impact of NPWT on SSI Odds ratios are shown with 95% confidence intervals. SSI surgical-site infection; NPWT, negative-pressure wound therapy.

Across 45 RCTs, NPWT significantly reduced SSI (OR 0.53, 95% confidence interval (c.i.) 0.42 to 0.66; *I*² = 50%) (*Fig. 3*). Among studies limited to abdominal surgery alone, NPWT reduced SSI (OR 0.52, 0.42 to 0.66; *I*^2^ = 50%), but no difference was found in open cardiac surgery (OR 0.44, 0.00 to 45.25; *I*^2^ = 66%, 3 RCTs).

**Fig. 4:**
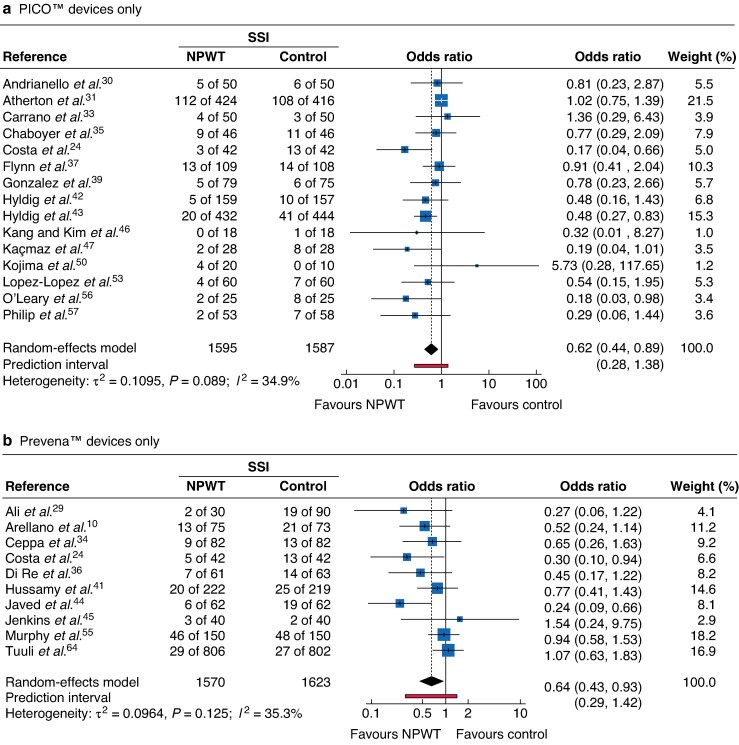
Forest plot showing meta-analysis of impact of NPWT on SSI according to specific device type **a** PICO™ devices only, **b** Prevena™ devices only. Odds ratios are shown with 95% confidence intervals. SSI surgical-site infection; NPWT, negative-pressure wound therapy.

Device-specific subgroup analyses demonstrated a reduction in SSI with both PICO™ (OR 0.62, 0.44 to 0.89; *I*² = 34.9%) and Prevena™ (OR 0.64, 0.43 to 0.93; *I*² = 35.3%), (*Fig. 4*). These findings suggest that the observed benefit represents a class effect of NPWT rather than superiority of a specific device. Trials using other proprietary or non-commercial devices were fewer, and limited data precluded meta-analysis.

**Fig. 5:**
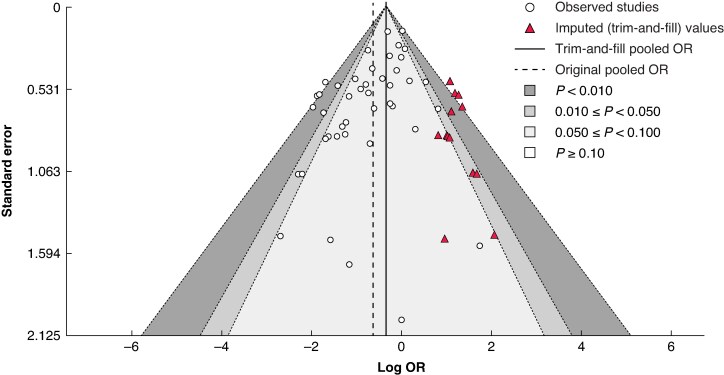
Funnel plot: original *versus* trim-and-fill method Contour lines show significance levels. Original pooled odds ratio (OR) 0.53; trim-and-fill pooled OR 0.70.

Assessment of publication bias suggested the presence of small-study effects. Visual inspection of the funnel plot revealed asymmetry, with a relative absence of small negative studies (*Fig. 5*). Egger’s regression test was statistically significant (*z* = −3.9465, *P* < 0.001), consistent with potential publication bias. Application of the trim-and-fill method suggested that 13 studies (standard error 4.3576) may be missing from the right-hand side of the funnel plot. After adjustment, the pooled OR shifted from 0.53 (0.42 to 0.66) to 0.70 (0.54 to 0.90), indicating that, although NPWT remained associated with a statistically significant reduction in SSI, the magnitude of benefit may be overestimated because of publication bias.

### Further wound complications

**Fig. 6:**
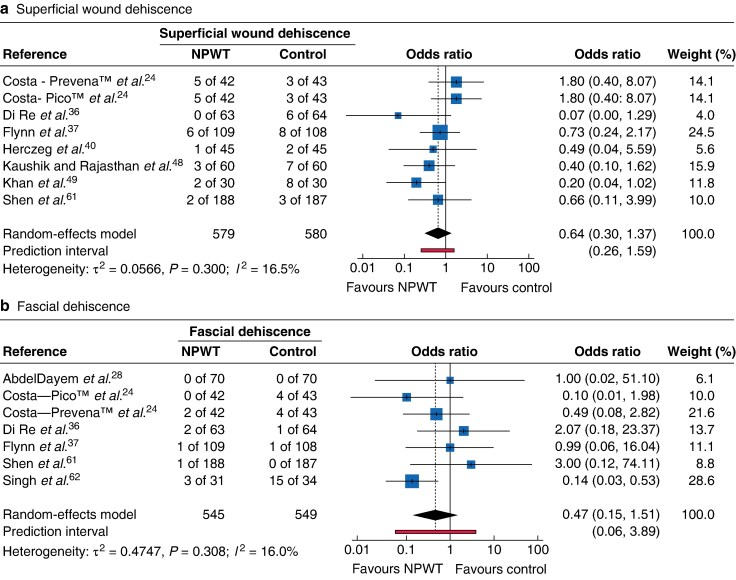
Forest plot showing meta-analysis of impact of NPWT on superficial wound dehiscence and fascial dehiscence **a** Superficial wound dehiscence**, b** fascial dehiscence. Odds ratios are shown with 95% confidence intervals. NPWT, negative-pressure wound therapy.

Despite significant differences in wound infection rates, use of NPWT had no effect on rates of organ/space infection (OR 0.92, 95% c.i. 0.67 to 1.25; 11 RCTs) (*Fig. S2*) or superficial or deep wound dehiscence (*Fig.* 6). This is perhaps reflective of the definition of SSI in most studies, with use of clinical assessment rather than objective endpoints such as wound culture results (*Table S4*).

### Length of hospital stay

**Fig. 7:**
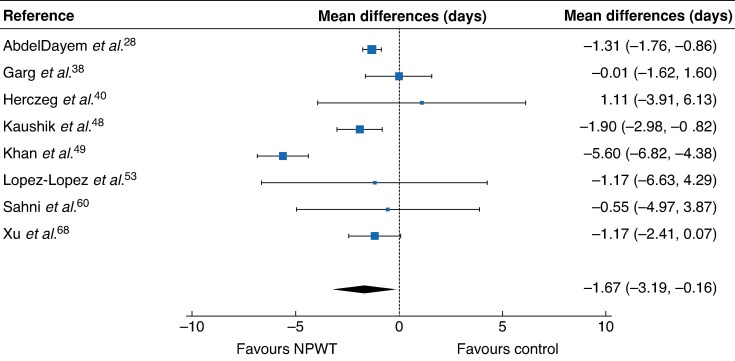
Forest plot showing meta-analysis of impact of NPWT on length of hospital stay Mean differences between negative-pressure wound therapy and control are shown with 95% confidence intervals. A random-effects model was used for meta-analysis.

Length of hospital stay was shorter among patients receiving NPWT (mean difference −1.67 (95% c.i. −3.19 to −0.16) days; 8 RCTs), with low heterogeneity (*Fig. 7*).

### Reoperation

**Fig. 8:**
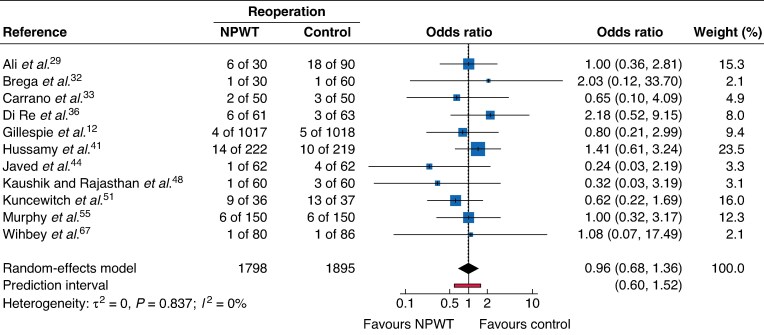
Forest plot showing meta-analysis of impact of NPWT on reoperation rate Odds ratios are shown with 95% confidence intervals. NPWT, negative-pressure wound therapy.

Use of NPWT had no impact on reoperation rates (OR 0.96, 95% c.i. 0.68 to 1.36; 11 RCTs), with low heterogeneity (*Fig. 8*).

### Other outcomes

Eight studies reported patient pain scores using a variety of questionnaires and scales, including a visual analogue scale and the patient-specific activity scale. Overall, pain scores were similar at 30 days after operation when reported. Earlier in the recovery period, between 3 and 7 days, pain scores appeared favourable towards use of NPWT (Atherton *et al*., and Manik *et al*.). However, several studies reported no difference in pain scores, including Ruhstaller *et al*. and Flynn *et al*.

QoL scores were assessed and reported in 6 studies using a variety of questionnaires, including the 12-item Short Form (SF-12^®^) and EQ-5D-5L™ (EuroQoL Group, Rotterdam, the Netherlands) surveys, and patient and observer scar assessment scale. Overall, there did not appear to be any clear difference between the two groups. For instance, using both SF-12 and^®^ EQ-5D-5L™, Atherton *et al*.^31^ found no difference in QoL scores between the two groups. This was supported by similar QoL scores (*P* = 0.89) reported by O’Leary *et al.*, Hyldig *et al.* (*P* = 0.319), and Lopez-Lopez *et al.* (*P* = 0.231). In contrast, Tuuli *et al.* reported significantly higher patient satisfaction scores in the NPWT group at discharge (*P* < 0.001). However, this difference was no longer significant compared with the control group on postoperative day 30 (*P* = 0.070).

## Discussion

This systematic review and meta-analysis has demonstrated the benefit and safety profile of NPWT for closed abdominal surgical wounds. Across 45 RCTs including over 12 000 patients, use of NPWT halved the risk of SSI and shortened hospital stay compared with standard dressings. These findings were consistent across commonly used commercial devices, supporting the potential for broader adoption in abdominal surgery. By contrast, evidence in open cardiothoracic surgery remains limited, and no significant benefit was observed for organ/space infection, fascial dehiscence, or reoperation.

This study has several strengths. First, inclusion was restricted to RCTs, thereby reducing the influence of confounding and selection bias that often complicates observational or retrospective series. Second, the search strategy was comprehensive and the statistical methodology robust, with predefined subgroup analyses by device type and adherence to a prospectively registered protocol. Third, given the heterogeneity of surgical populations and study designs, the use of random-effects modelling was appropriate and increases the generalizability of findings across surgical contexts. Finally, given the findings published by Groenen *et al*. (2023), assessing all RCTs in prevention of SSI across all surgical specialties, the present study reports results favouring NPWT for SSI prevention, with the additional components of publication bias assessment, and device-specific subgroup analyses.

Publication bias for the primary outcome (*P* < 0.001) suggests that smaller studies reported larger beneficial effects of NPWT than larger studies, perhaps reflecting selective publication of positive findings (or suppression of negative results) from smaller trials. This could mean the pooled OR of 0.53 may be overestimated owing to under-reporting of negative trials. To further explore this, the trim-and-fill method was applied, which estimated that 13 studies may be missing from the right side of the funnel plot. After imputing these hypothetical trials, the pooled OR decreased from 0.54 (95% c.i. 0.43 to 0.69) to 0.70 (0.54 to 0.90). Importantly, the association between NPWT and reduced odds of SSI remained statistically significant, but the magnitude of benefit was reduced, indicating that the effect size reported in the unadjusted analysis is likely to be somewhat overestimated. The pooled results must therefore be interpreted with caution, as negative trials may remain unpublished.

Different types of abdominal surgery (for example colorectal, hepatobiliary, bariatric, obstetric) have different baseline risks of SSI. However, meta-analyses of individual surgical subspecialties can make it difficult to assess whether hospital boards should invest in such technology to be used across surgical specialties, rather than limiting a technology to one or a few specialties^69^. Although subspecialty-specific analyses can be informative, decisions regarding investment in NPWT technology are often made at the hospital or health system level, necessitating pooled evaluation across surgical types.

Furthermore, NPWT effectiveness may vary by type of incision (midline, Pfannenstiel, bisubcostal), contamination level, or patient co-morbidities. However the present meta-analysis included a range of incision types and procedure- or patient-related confounders. Over half of the studies used US Centers for Disease Control and Prevention criteria for diagnosis of SSI. The remainder utilized clinical assessment and subjective diagnosis, perhaps also relying on biochemical and/or clinical parameters. However, the absence of wound culture results from the studies may suggest an overestimation of the prevalence of SSI.

The present analysis necessarily combined a wide range of incision types and patient-related factors, which may have diluted signals of benefit in subgroups at highest risk. There was also variation in definition of SSI, follow-up duration, type of NPWT device used, control dressing use, and adherence to NPWT protocol as advised by the manufacturer. Finally, trial follow-up was generally limited to the early postoperative period. One of the longer-term complications of a SSI in the late postoperative phase is the development of incisional hernia^70^. However, limited follow-up duration in the trials analysed precluded the detection and reporting of this important long-term outcome.

Industry funding was common among the included trials, particularly for proprietary systems such as Prevena™ and PICO™. Although device-specific analyses demonstrated consistent benefit, the role of manufacturer sponsorship raises concerns regarding selective publication, reporting bias, and emphasis on device-specific outcomes. Indeed, an analysis of 332 historical RCTs by Bhandari *et al*.^71^ in 2004 showed that industry funding was directly associated with a statistically significant (pro-industry) result (OR 1.9, 95% c.i. 1.3 to 3.5). Independent, investigator-led trials remain critical to confirming these findings in routine practice. Trials in this study also showed variation in the control dressing utilized. There was limited investigator blinding, and no patient blinding, for example through the use of a placebo or sham device. The control dressings used were referred to as ‘standard’ or ‘surgeon’s preference’ or ‘simple gauze’ in most trials. Advanced non-suction dressings such as the Smith & Nephew honeycomb dressing was used in one trial (Jenkins *et al*.).

Guidelines for SSI prevention have been cautious historically in recommending NPWT for closed incisions, largely because trial evidence was limited^69^. The present findings, showing a clear reduction in SSI across a broad range of abdominal procedures, strengthen the rationale for guideline endorsement of NPWT in high-risk patients. At present, many centres adopt a selective approach, targeting NPWT to patients with high-risk characteristics or complex procedures. This aligns with the present findings that the greatest benefit is likely to accrue in such populations, and reflects the need for judicious resource allocation given the higher upfront cost of NPWT devices. Information on the cost-effectiveness of these devices in scarce in the literature. According to PICO™ base-case economic modelling, PICO™ dressings are cost-saving by around £101 per patient compared with standard dressings^72^.

Commercially available systems such as Prevena™ are marketed for use in patients with multiple risk factors and broad definitions of high surgical risk. Suitability criteria commonly include at least two of the following: BMI > 35 kg/m^2^, diabetes mellitus, active smoking, malignancy or chemotherapy, immunodeficiency, chronic kidney disease, chronic obstructive pulmonary disease, advanced age (≥ 75 years), or malnutrition. High-risk procedures include emergency or revisional surgery, prolonged operating time, contaminated or traumatic wounds, incisions under high tension, or multiple simultaneous incisions. These thresholds broadly align with the populations represented in the present meta-analysis and may help guide rational deployment of NPWT in practice. Such risk categories are also replicated in national guidance. For instance, the UK National Institute for Health and Care Excellence Medical Technology Guidance 43^73^ reflects this, and goes further to recommend PICO™ dressings for closed surgical wounds, citing reduction in SSI and cost-effectiveness compared with conventional wound dressings alone.

Future research should focus on several areas. First, adequately powered, pragmatic RCTs across selected surgical populations are required to validate efficacy in real-world settings and quantify the absolute risk reduction across baseline SSI observation, especially in the eclectic emergency setting. Second, independent health economic analyses are urgently needed, as the incremental cost-effectiveness of NPWT remains uncertain outside high-risk subgroups. This is essential in informing the use of this resource in the public sector, such as in the National Health Service. Third, longer follow-up is essential to determine whether early reductions in SSI translate into fewer late complications such as incisional hernia. Finally, comparative trials between different NPWT systems, and between NPWT and enhanced conventional dressings, would clarify whether benefits are device-specific or generalizable to the class.

The present analysis has shown that NPWT halves the risk of SSI following open abdominal surgery and modestly reduces hospital stay, with consistent benefits across commercial devices. Evidence for other outcomes remains inconclusive, and the signal of publication bias underscores the need for independent confirmatory studies. These findings support the selective use of NPWT in high-risk patients and procedures, while highlighting the importance of cost-effectiveness analyses and longer-term outcome data in guiding routine implementation.

## Supplementary Material

zrag027_Supplementary_Data

## Data Availability

All data are available publicly in the original published article.

## References

[1] Carney MJ, Weissler JM, Fox JP, Tecce MG, Hsu JY, Fischer JP. Trends in open abdominal surgery in the United States—observations from 9 950 759 discharges using the 2009–2013 National Inpatient Sample (NIS) datasets. Am J Surg 2017;214:287–292 28202162 10.1016/j.amjsurg.2017.01.001

[2] Melly L, Torregrossa G, Lee T, Jansens JL, Puskas JD. Fifty years of coronary artery bypass grafting. J Thorac Dis 2018;10:1960–1967 29707352 10.21037/jtd.2018.02.43 PMC5906252

[3] Khoury AL, Patel S, Ngeve S, Doughty K, Wilson HK, Caranasos TG. FlatWire sternal closure system technique for median sternotomy closure. J Thorac Dis 2023;15:5037–5040 37868890 10.21037/jtd-23-110 PMC10586947

[4] Deerenberg EB, Henriksen NA, Antoniou GA, Antoniou SA, Bramer WM, Fischer JP et al Updated guideline for closure of abdominal wall incisions from the European and American Hernia Societies. Br J Surg 2022;109:1239–1250 36026550 10.1093/bjs/znac302 PMC10364727

[5] Zabaglo M, Leslie SW, Sharman T. Postoperative Wound Infections. Treasure Island: StatPearls Publishing, 2025 32809368

[6] Shepard J, Ward W, Milstone A, Carlson T, Frederick J, Hadhazy E et al Financial impact of surgical site infections on hospitals: the hospital management perspective. JAMA Surg 2013;148:907–914 23965750 10.1001/jamasurg.2013.2246

[7] Zimlichman E, Henderson D, Tamir O, Franz C, Song P, Yamin CK et al Health care-associated infections: a meta-analysis of costs and financial impact on the US health care system. JAMA Intern Med 2013;173:2039–2046 23999949 10.1001/jamainternmed.2013.9763

[8] Bucataru A, Balasoiu M, Ghenea AE, Zlatian OM, Vulcanescu DD, Horhat FG et al Factors contributing to surgical site infections: a comprehensive systematic review of etiology and risk factors. Clin Pract 2023;14:52–68 38248430 10.3390/clinpract14010006 PMC10801486

[9] Marzoug OA, Anees A, Malik EM. Assessment of risk factors associated with surgical site infection following abdominal surgery: a systematic review. BMJ Surg Interv Health Technologies 2023;5:e000182 10.1136/bmjsit-2023-000182 PMC10387634 37529828

[10] Arellano M, Barragán Serrano C, Guedea M, Garcia Pérez JC, Sanz Ortega G, Guevara-Martinez J et al Surgical wound complications after colorectal surgery with single-use negative-pressure wound therapy *versus* surgical dressing over closed incisions: a randomized controlled trial. Adv Skin Wound Care 2021;34:657–661 34175866 10.1097/01.ASW.0000756512.87211.13

[11] Anestiadou E, Stamiris S, Ioannidis O, Symeonidis S, Bitsianis S, Bougioukas K et al Comparison of negative pressure wound therapy systems and conventional non-pressure dressings on surgical site infection rate after stoma reversal: systematic review and meta-analysis of randomized controlled trials. J Clin Med 2025;14:1654 40095625 10.3390/jcm14051654 PMC11900534

[12] Gillespie BM, Webster J, Ellwood D, Thalib L, Whitty JA, Mahomed K et al Closed incision negative pressure wound therapy *versus* standard dressings in obese women undergoing caesarean section: multicentre parallel group randomised controlled trial. BMJ 2021;373:n893 33952438 10.1136/bmj.n893 PMC8097312

[13] Feier CVI, Gaborean V, Faur IF, Vonica RC, Faur AM, Rus VI et al A systematic review of closed-incision negative-pressure wound therapy for hepato-pancreato-biliary surgery: updated evidence, context, and clinical implications. J Clin Med 2025;14:5191 40806812 10.3390/jcm14155191 PMC12347151

[14] Groenen H, Jalalzadeh H, Buis DR, Dreissen YEM, Goosen JHM, Griekspoor M et al Incisional negative pressure wound therapy for the prevention of surgical site infection: an up-to-date meta-analysis and trial sequential analysis. eClinicalMedicine 2023;62:102105 37538540 10.1016/j.eclinm.2023.102105 PMC10393772

[15] STARSurg Collaborative, EuroSurg Collaborative. Association between multimorbidity and postoperative mortality in patients undergoing major surgery: a prospective study in 29 countries across Europe. Anaesthesia 2024;79:945–956 39101671 10.1111/anae.16324

[16] Cochrane . *Cochrane Handbook for Systematic Reviews of Interventions*. https://training.cochrane.org/handbook (accessed 8 June 2025)

[17] Page MJ, McKenzie JE, Bossuyt PM, Boutron I, Hoffmann TC, Mulrow CD et al The PRISMA 2020 statement: an updated guideline for reporting systematic reviews. BMJ 2021;372:n71 33782057 10.1136/bmj.n71 PMC8005924

[18] PROSPERO. *Negative Pressure Wound Therapy After Open Thoracoabdominal Surgery: A Systematic Review and Meta-Analysis of Randomised Trials.* https://www.crd.york.ac.uk/PROSPERO/view/CRD420251010516 (accessed 8 June 2025)

[19] Bramer WM, Giustini D, de Jonge GB, Holland L, Bekhuis T. De-duplication of database search results for systematic reviews in EndNote. J Med Libr Assoc 2016;104:240–243 27366130 10.3163/1536-5050.104.3.014 PMC4915647

[20] Cochrane . *RoB 2: A Revised Cochrane Risk-of-bias Tool for Randomized Trials*. https://methods.cochrane.org/bias/resources/rob-2-revised-cochrane-risk-bias-tool-randomized-trials (accessed 25 October 2024)

[21] Clavien PA, Barkun J, de Oliveira ML, Vauthey JN, Dindo D, Schulick RD et al The Clavien–Dindo classification of surgical complications: five-year experience. Ann Surg 2009;250:187–196 19638912 10.1097/SLA.0b013e3181b13ca2

[22] Egger M, Smith GD, Schneider M, Minder C. Bias in meta-analysis detected by a simple, graphical test. BMJ 1997;315:629–634 9310563 10.1136/bmj.315.7109.629 PMC2127453

[23] DerSimonian R, Kacker R. Random-effects model for meta-analysis of clinical trials: an update. Contemp Clin Trials 2007;28:105–114 16807131 10.1016/j.cct.2006.04.004

[24] Costa MJ, Martins MF, Lages RR, Gonçalves ÁL, Armas IS, Almeida JI et al Optimizing closed incision negative pressure wound therapy in emergency laparotomy (OPTIWOUND): a multi-arm randomized prospective trial. Surg Gastroenterol Oncol 2024;29:131

[25] Science Direct . *Yates Continuity Correction—An Overview*. https://www.sciencedirect.com/topics/medicine-and-dentistry/yates-continuity-correction (accessed 24 October 2024)

[26] Moher D, Hopewell S, Schulz KF, Montori V, Gøtzsche PC, Devereaux PJ et al CONSORT 2010 explanation and elaboration: updated guidelines for reporting parallel group randomised trials. BMJ 2010;340:c869–c869 20332511 10.1136/bmj.c869 PMC2844943

[27] Pijls BG . The table I fallacy: *P* values in baseline tables of randomized controlled trials. J Bone Joint Surg Am 2022;104:e71 35230980 10.2106/JBJS.21.01166

[28] AbdelDayem AM, Nashed GA, Balamoun HA, Mostafa MS. Effectiveness of 3-day prophylactic negative pressure wound therapy on closed abdominal incisions in the prevention of wound complications: a randomized controlled trial. J Gastrointest Surg 2023;27:1702–1709 37407900 10.1007/s11605-023-05752-3

[29] Ali RM, El Fattah ARA, Abdelhady WA, Morsy AM, Fahmy AM, Harb OA et al Use of prophylactic closed-incision negative-pressure therapy (CINPT) is associated with reduced surgical-site infections in patients undergoing open abdominal surgeries during the COVID-19 pandemic. Egypt J Surg 2021;40:850

[30] Andrianello S, Landoni L, Bortolato C, Iudici L, Tuveri M, Pea A et al Negative pressure wound therapy for prevention of surgical site infection in patients at high risk after clean-contaminated major pancreatic resections: a single-center, phase 3, randomized clinical trial. Surgery 2021;169:1069–1075 33257037 10.1016/j.surg.2020.10.029

[31] SUNRRISE Trial Study Group . Negative pressure dressings to prevent surgical site infection after emergency laparotomy: the SUNRRISE randomized clinical trial. JAMA 2025;333:853–863 39869330 10.1001/jama.2024.24764 PMC11773404

[32] Brega C, Calvi S, Albertini A. Use of a negative pressure wound therapy system over closed incisions option in preventing post-sternotomy wound complications. Wound Repair Regen 2021;29:848–852 33780088 10.1111/wrr.12914

[33] Carrano FM, Maroli A, Carvello M, Foppa C, Sacchi M, Crippa J et al Negative-pressure wound therapy after stoma reversal in colorectal surgery: a randomized controlled trial. BJS Open 2021;5:zrab116 34904647 10.1093/bjsopen/zrab116 PMC8669787

[34] Ceppa EP, Kim RC, Niedzwiecki D, Lowe ME, Warren DA, House MG et al Closed incision negative pressure therapy to reduce surgical site infection in high-risk gastrointestinal surgery: a randomized controlled trial. J Am Coll Surg 2023;236:698 36728375 10.1097/XCS.0000000000000547

[35] Chaboyer W, Anderson V, Webster J, Sneddon A, Thalib L, Gillespie BM. Negative pressure wound therapy on surgical site infections in women undergoing elective caesarean sections: a pilot RCT. Healthcare 2014;2:417–428 27429285 10.3390/healthcare2040417 PMC4934567

[36] Di Re AM, Wright D, Toh JWT, El-Khoury T, Pathma-nathan N, Gosselink MP et al Surgical wound infection prevention using topical negative pressure therapy on closed abdominal incisions—the ‘SWIPE IT’ randomized clinical trial. J Hosp Infect 2021;110:76–83 33516795 10.1016/j.jhin.2021.01.013

[37] Flynn J, Choy A, Leavy K, Connolly L, Alards K, Ranasinha S et al Negative pressure dressings (PICO^TM^) on laparotomy wounds do not reduce risk of surgical site infection. Surg Infect 2020;21:231–238 10.1089/sur.2019.078 31618115

[38] Garg A, Jayant S, Gupta AK, Bansal LK, Wani A, Chaudhary P. Comparison of closed incision negative pressure wound therapy with conventional dressing for reducing wound complications in emergency laparotomy. *Pol Przegl Chir* 2021;93:1–5. doi: 10.5604/01.3001.0014.9759 34552028

[39] Gonzalez MG, Barske ME, Kjellsson KB, Saboda K, Reed HA, Hill MG. Topical negative pressure wound therapy to prevent wound complications following caesarean delivery in high-risk obstetric patients: a randomised controlled trial. Aust N Z J Obstet Gynaecol 2023;63:516–520 37140175 10.1111/ajo.13675

[40] Herczeg A, Szijártó A, Fülöp A, Varga K, Marton J, Lóderer Z et al Prophylactic negative pressure wound therapy reduces superficial surgical site infection risk of emergency surgery patients: results of a multicenter randomised prospective clinical trial. Int Wound J 2025;22:e70718 40605477 10.1111/iwj.70718 PMC12223186

[41] Hussamy DJ, Wortman AC, McIntire DD, Leveno KJ, Casey BM, Roberts SW. Closed incision negative pressure therapy in morbidly obese women undergoing cesarean delivery: a randomized controlled trial. Obstet Gynecol 2019;134:781 31503147 10.1097/AOG.0000000000003465

[42] Hyldig N, Vinter C, Kruse M, Mogensen O, Bille C, Sorensen J et al Prophylactic incisional negative pressure wound therapy reduces the risk of surgical site infection after caesarean section in obese women: a pragmatic randomised clinical trial. BJOG 2019;126:628–635 30066454 10.1111/1471-0528.15413 PMC6586160

[43] Hyldig N, Möller S, Joergensen JS, Bille C. Clinical evaluation of scar quality following the use of prophylactic negative pressure wound therapy in obese women undergoing cesarean delivery: a trial-based scar evaluation. Ann Plast Surg 2020;85:e59 32657852 10.1097/SAP.0000000000002468

[44] Javed AA, Teinor J, Wright M, Ding D, Burkhart RA, Hundt J et al Negative pressure wound therapy for surgical-site infections: a randomized trial. Ann Surg 2019;269:1034–1040 31082899 10.1097/SLA.0000000000003056

[45] Jenkins S, Komber M, Mattam K, Briffa N. Negative pressure wound therapy in patients with diabetes undergoing left internal thoracic artery harvest: a randomized control trial. J Thorac Cardiovasc Surg 2024;167:256–268 35550716 10.1016/j.jtcvs.2022.01.060

[46] Kang SI, Kim S. The effectiveness of negative-pressure wound therapy for wound healing after stoma reversal: a randomized control study. Ann Surg Treat Res 2023;105:126–132 37693285 10.4174/astr.2023.105.3.126 PMC10485349

[47] Kaçmaz HY, Baser M, Sozuer EM. Effect of prophylactic negative-pressure wound therapy for high-risk wounds in colorectal cancer surgery: a randomized controlled trial. Adv Skin Wound Care 2022;35:597–603 36264751 10.1097/01.ASW.0000874168.60793.10

[48] Kaushik DV, Rajasthan A. Evaluation of surgical site infection rates with closed-incision negative pressure wound therapy *versus* standard dressing in colorectal surgery. Int J Life Sci 2025;14

[49] Khan M . Effectiveness of closed incision negative pressure therapy compared to conventional moist dressing after laparotomy for peritonitis: a randomized controlled trial. J Popul Ther Clin Pharmacol 2025:962–967

[50] Kojima K, Goto M, Nagashima Y, Saito Y, Kawai M, Takebe S et al Effectiveness of negative pressure wound therapy for the wound of ileostomy closure: a multicenter, phase II randomized controlled trial. BMC Surg 2021;21:442 34963451 10.1186/s12893-021-01446-2 PMC8713411

[51] Kuncewitch MP, Blackham AU, Clark CJ, Dodson RM, Russell GB, Levine EA et al Effect of negative pressure wound therapy on wound complications post-pancreatectomy. Am Surg 2019;85:1–7 30760337 PMC6743488

[52] Li PY, Yang D, Liu D, Sun SJ, Zhang LY. Reducing surgical site infection with negative-pressure wound therapy after open abdominal surgery: a prospective randomized controlled study. Scand J Surg 2017;106:189–195 27609528 10.1177/1457496916668681

[53] Lopez-Lopez V, Hiciano-Guillermo A, Martinez-Alarcon L, Delegido A, Alconchel F, Pons JA et al Postoperative negative-pressure incision therapy after liver transplant (PONILITRANS study): a randomized controlled trial. Surgery 2023;173:1072–1078 36549975 10.1016/j.surg.2022.11.011

[54] Manik M, Anandhi A, Sureshkumar S, Keerthi AR, Sudharshan M, Kate V. Prophylactic negative pressure wound therapy in reducing surgical site infections in closed abdominal incision: a randomized controlled trial. Adv Wound Care 2024;13:123–130 10.1089/wound.2023.0052 37646410

[55] Murphy P, Lee K, Dubois L, DeRose G, Forbes T, Power A. Negative pressure wound therapy for high-risk wounds in lower extremity revascularization: study protocol for a randomized controlled trial. Trials 2015;16:504 26537879 10.1186/s13063-015-1026-1 PMC4634141

[56] O’Leary DP, Peirce C, Anglim B, Burton M, Concannon E, Carter M et al Prophylactic negative pressure dressing use in closed laparotomy wounds following abdominal operations: a randomized, controlled, open-label trial: the P.I.C.O. *trial.* Ann Surg 2017;265:1082–1086 27926575 10.1097/SLA.0000000000002098

[57] Philip EF, Rajandram R, Zuber M, Khong TL, Roslani AC. Prophylactic PICO^◊^ dressing shortens wound dressing requirements post emergency laparotomy (EL-PICO^◊^ trial). World J Emerg Surg 2024;19:38 39578859 10.1186/s13017-024-00560-9 PMC11583525

[58] Rashed A, Csiszar M, Beledi A, Gombocz K. The impact of incisional negative pressure wound therapy on the wound healing process after midline sternotomy. Int Wound J 2020;18:95–102 33236860 10.1111/iwj.13497 PMC7948622

[59] Ruhstaller K, Downes KL, Chandrasekaran S, Srinivas S, Durnwald C. Prophylactic wound vacuum therapy after cesarean section to prevent wound complications in the obese population: a randomized controlled trial (the ProVac study). Am J Perinatol 2017;34:1125–1130 28704847 10.1055/s-0037-1604161 PMC5983905

[60] Sahni K, Hosamani S, Ghuliani D, Baisoya S. Evaluation of negative pressure dressings for closed surgical incisions in decreasing surgical site infections after emergency laparotomy: a randomized controlled study. Cureus 2024;16:e67500 39310489 10.7759/cureus.67500 PMC11416178

[61] Shen P, Blackham AU, Lewis S, Clark CJ, Howerton R, Mogal HD et al Phase II randomized trial of negative-pressure wound therapy to decrease surgical site infection in patients undergoing laparotomy for gastrointestinal, pancreatic, and peritoneal surface malignancies. J Am Coll Surg 2017;224:726–737 28088597 10.1016/j.jamcollsurg.2016.12.028 PMC5498990

[62] Singh H, Avudaiappan M, Kharel J, Irrinki S, Kumar H, Savlania A et al Negative pressure wound therapy *versus* standard care for incisional laparotomy subcutaneous wounds in gastrointestinal perforations: a randomized controlled study. Surgery 2023;174:291–295 37183134 10.1016/j.surg.2023.04.018

[63] Tiang T, Behrenbruch C, Noori J, Lam D, Bhamidipaty M, Johnston M et al Prophylactic negative pressure wound therapy to improve wound healing rates following ileostomy closure: a randomized controlled trial. ANZ J Surg 2024;94:1627–1633 38525845 10.1111/ans.18941

[64] Tuuli MG, Liu J, Tita ATN, Longo S, Trudell A, Carter EB et al Effect of prophylactic negative pressure wound therapy *vs* standard wound dressing on surgical-site infection in obese women after cesarean delivery. JAMA 2020;324:1180–1189 32960242 10.1001/jama.2020.13361 PMC7509615

[65] Whitty JA, Wagner AP, Kang E, Ellwood D, Chaboyer W, Kumar S et al Cost-effectiveness of closed incision negative pressure wound therapy in preventing surgical site infection among obese women giving birth by caesarean section: an economic evaluation (DRESSING trial). Aust N Z J Obstet Gynaecol 2023;63:673–680 37200473 10.1111/ajo.13677 PMC10952760

[66] Wierdak M, Pisarska-Adamczyk M, Wysocki M, Major P, Kołodziejska K, Nowakowski M et al Prophylactic negative-pressure wound therapy after ileostomy reversal for the prevention of wound healing complications in colorectal cancer patients: a randomized controlled trial. Tech Coloproctol 2021;25:185–193 33161523 10.1007/s10151-020-02372-w PMC7884579

[67] Wihbey KA, Joyce EM, Spalding ZT, Jones HJ, MacKenzie TA, Evans RH et al Prophylactic negative pressure wound therapy and wound complication after cesarean delivery in women with class II or III obesity: a randomized controlled trial. Obstet Gynecol 2018;132:377–384 29995726 10.1097/AOG.0000000000002744

[68] Xu DY, Bai BJ, Shan L, Wei HY, Lin DF, Wang Y et al Micro-power negative pressure wound technique reduces risk of incision infection following loop ileostomy closure. World J Gastrointest Surg 2024;16:186–195 38328332 10.4240/wjgs.v16.i1.186 PMC10845270

[69] Norman G, Shi C, Goh EL, Murphy EM, Reid A, Chiverton L et al Negative pressure wound therapy for surgical wounds healing by primary closure. Cochrane Database Syst Rev 2022; (4)CD009261 35471497 10.1002/14651858.CD009261.pub7 PMC9040710

[70] Hope WW, Tuma F. Incisional Hernia. Treasure Island: StatPearls Publishing, 2025. 28613766

[71] Bhandari M, Busse JW, Jackowski D, Montori VM, Schünemann H, Sprague S et al Association between industry funding and statistically significant pro-industry findings in medical and surgical randomized trials. CMAJ 2004;170:477–480 14970094 PMC332713

[72] National Institute for Health and Care Excellence. *PICO Negative Pressure Wound Dressings for Closed Surgical Incisions—Guidance*. https://www.nice.org.uk/guidance/mtg43/chapter/3-Evidence#cost-evidence (accessed 6 September 2025)

[73] National Institute for Health and Care Excellence. *PICO Negative Pressure Wound Dressings for Closed Surgical Incisions—Recommendations*. England, UK: NICE. https://www.nice.org.uk/guidance/MTG43/chapter/1-Recommendations (accessed 18 August 2025)

